# Differential gene expression in G-MDSC and neutrophils from renal cell carcinoma patients

**DOI:** 10.1186/2051-1426-2-S3-P216

**Published:** 2014-11-06

**Authors:** Jennifer Ko, Patricia A Rayman, Yu Yang, Banu Gopalan, James Finke

**Affiliations:** 1 Cleveland Clinic, Cleveland, OH, USA; 2 Cleveland Clinic Foundation, Cleveland, OH, USA; 3 Yorg Corporation, USA

## 

Myeloid-derived suppressor cells (MDSC), including undifferentiated, monocytic, and granulocytic subsets, are known to play a role in tumor progression via promotion of tumor invasion and angiogenesis, and suppression of Type 1 T cell responses. Elevated peripheral blood neutrophil counts have long been associated with poor outcome in multiple tumor types, and recently tumor-associated neutrophils (TAN) were shown to have similar properties as granulocytic MDSC (G-MDSC). The relationship between G-MDSC and neutrophils is unclear; although recent published data show that G-MDSC from tumor-bearing mice and activated neutrophils from non-tumor-bearing mice show differential expression of numerous genes by cDNA analysis. Here we have tried to dissect this relationship more deeply, in clear cell renal cell carcinoma (ccRCC) patients, by using microarray cDNA data to compare gene expression analysis in cancer patient G-MDSC and patient neutrophils. G-MDSC and neutrophils in patients were discriminated based on their density, and the ability of G-MDSC to co-precipitate with peripheral blood mononuclear cells. We found that genes upregulated in G-MDSC were mostly involved in protein translation initiation (EEF1A1, RPL15, LOC440595, RPL22, RPS3, and EIF4A1), chromatin remodeling (HNRNPR), and cell cycle, G1-S growth factor regulation through AP-1 signaling (JUN and PRKCB1). Additional upregulated genes of interest included DNAJA2, co-chaperone of Hsp70, which was recently implicated in Treg-mediated immune suppression, as well as the anti-apoptotic protein TMBIM6. Genes that were relatively upregulated in patient neutrophils compared to G-MDSC included those related to cell adhesion (FLOT2, TUBA4A, LLPH, and PCDHB9) and cellular response to external signals. These included S100A11 which, upon ligation with RAGE, leads to NFkB and MAPK activation involved in oxidative stress; FOXO4, involved in wound healing, stress response, and elaboration of anti-microbial peptides; VPS41, involved in lysosomal trafficking; and, finally, CFLAR, which is anti-apoptotic. The findings support the notion that G-MDSC in ccRCC patients represent relatively less mature neutrophils and neutrophil precursors which are actively cycling and making proteins, some of which may be necessary for their functional activity. Meanwhile, patient neutrophils which co-precipitate with red blood cells, appear to function mostly in response to stimulatory and adverse external signals, some of which are likely to be involved in the G-MDSC-like functions previously described for TAN. G-MDSC are likely to differentiate into the activated neutrophils described in cancer patients. Further studies will need to focus on the differences between those neutrophils found in cancer patients and those found in apparently healthy individuals.

